# Exercise intervention on sleep quality in Alzheimer’s disease: a systematic review and meta-analysis

**DOI:** 10.3389/fmed.2026.1796892

**Published:** 2026-05-08

**Authors:** Zelong Tan, Yichen Jin, Zhipei Niu

**Affiliations:** College of Sports Leisure and Tourism, Beijing Sport University, Beijing, China

**Keywords:** Alzheimer’s disease, exercise, meta-analysis, sleep quality, systematic review

## Abstract

**Background:**

Patients with Alzheimer’s disease (AD) are often accompanied by severe sleep disorders, which exacerbate with disease progression. Currently, pharmacological treatments have limitations, and the effect of exercise as a non-pharmacological intervention on improving sleep quality in AD patients requires systematic evaluation.

**Methods:**

This study systematically searched six databases up to December 31, 2025, and included randomized controlled trials (RCTs) investigating the effects of exercise interventions on sleep quality in patients with Alzheimer’s disease. Two researchers independently completed literature screening, data extraction, and risk of bias assessment. Data analysis and literature quality evaluation were performed using Review Manager 5.4 software, and effect sizes were pooled using standardized mean differences (SMD).

**Results:**

A total of 12 RCTs involving 893 patients were finally included. Meta-analysis results showed that exercise interventions significantly improved patients’ subjective sleep quality, with a pooled effect size (SMD) of −0.81 for the Pittsburgh Sleep Quality Index (PSQI) score (95%CI: −1.55 to −0.06, *p* = 0.030). However, regarding objective sleep indicators, the improving effects of exercise on sleep efficiency (SMD = −0.23, 95%CI: −0.98 to 0.52, *p* = 0.850) and total sleep duration (SMD = 0.25, 95%CI: −0.53 to 1.03, *p* = 0.530) did not reach statistical significance. Subgroup analyses showed that the intervention effects were more significant in the subgroup with baseline PSQI > 10 (SMD = −1.92, 95% CI: −3.61 to −0.24, *p* = 0.03), the subgroup with single exercise duration ≥ 1 h (SMD = −1.34, 95% CI: −2.65 to −0.02, *p* = 0.05), and the aerobic exercise intervention subgroup (SMD = −1.34, 95% CI: −2.65 to −0.02, *p* = 0.05).

**Conclusion:**

Exercise interventions may improve subjective sleep quality in patients with Alzheimer’s disease, with potentially greater benefits observed in those with more severe baseline sleep disturbances or exercise sessions lasting ≥1 h; however, their effects on objective sleep outcomes have not yet been fully established. Further large-scale studies with more rigorous methodology and combined subjective and objective assessments are needed to clarify the true benefits of exercise on sleep in patients with Alzheimer’s disease and to determine the optimal intervention regimen.

**Systematic review registration:**

https://www.crd.york.ac.uk/PROSPERO/view/CRD420251270397.

## 1 Introduction

Alzheimer’s disease (AD) is a progressive neurodegenerative disorder and the most common form of dementia (1). According to a 2025 report by the Alzheimer’s Association, the number of AD patients aged 65 and older in the United States has surpassed 7.2 million, with projections indicating this figure will rise to over 12.7 million by 2050. Notably, between 2000 and 2022,deaths from heart disease in the United States decreased by 2.1%,while deaths from Alzheimer’s disease surged by 142% (2). The core symptom of this disease is progressive cognitive decline, primarily manifesting as memory loss (3), disorientation, impaired language and executive function, severely affecting patients’ social functioning and daily living abilities (4, 5). Currently, Alzheimer’s disease remains an irreversible neurological disorder (6). Therefore, there is an urgent need to develop effective prevention and treatment strategies to delay or halt its progression, thereby alleviating its growing socioeconomic burden (4, 7).

Research indicates that the prevalence of sleep disorders among AD patients reaches as high as 45% (8, 9), with symptoms including disrupted sleep architecture, altered sleep–wake rhythms, and changes in sleep breathing patterns (10, 11). Pathologically, sleep disturbances can trigger systemic and central nervous system inflammation, neurophysiological alterations, and increased accumulation of tau and beta-amyloid proteins in the brain, potentially driving the onset and progression of AD (12, 13). Furthermore, the decline in sleep quality is also associated with weakened connectivity between the functions of specific brain regions and cognition-related brain networks (14). These brain regions and their connections are also typically involved in the cognitive decline process of AD and other neurodegenerative disorders (15).

However, in terms of pharmacotherapy, there is currently a lack of specific and reliable drugs for AD. Multiple research evidences have shown that some commonly used medications such as cholinesterase inhibitors may increase the risk of adverse events in patients (16). Benzodiazepines, commonly used to improve sleep in patients with AD, are associated with a variety of side effects, including daytime drowsiness, rebound insomnia after drug discontinuation, confusion, amnesia, and an increased risk of falls (17). Although atypical antipsychotics can be used to manage behavioral symptoms, they may accelerate cognitive decline (18). Given the side effects and risks associated with pharmacological interventions, guidelines from institutions such as the National Institute for Health and Care Excellence (NICE) recommend non-pharmacological treatments as the first-line option (19). Among these, physical exercise, as an important non-pharmacological intervention, has been confirmed by a number of studies to not only improve cardiovascular function and alleviate mental health problems such as anxiety and depression, but also exert a positive effect on the improvement of cognitive function (20–22).

Existing evidence indicates that various forms of exercise, including combined training, resistance training, and aerobic training, can improve sleep quality in patients with Alzheimer’s disease. However, the generalizability of these conclusions and the depth of mechanistic exploration remain very limited. Veronese et al. reported in a cross-sectional study that exercise helps alleviate depressive symptoms and sleep disturbances in this population and proposed that it could serve as an adjunctive treatment (1). Nevertheless, the cross-sectional design inherently limits causal inference, and the underlying mechanisms lack validation from longitudinal experiments. Shih et al. also found that longer weekly walking duration and walking with relatives were associated with better sleep outcomes (23). However, these findings relied on subjective sleep reports and did not adequately control for potential confounders such as social interaction and disease severity. Regarding sleep architecture, the regulatory effects of exercise on the sleep microstructure of patients with Alzheimer’s disease have not been fully verified. Although Ahn and Kim observed an increase in rapid eye movement (REM) sleep duration after exercise intervention in patients with amnestic mild cognitive impairment, it remains unclear whether similar changes occur in patients with typical Alzheimer’s disease and whether such changes translate into cognitive benefits (24). A study by Zhang et al. demonstrated that exercise significantly improved sleep quality and total sleep time in patients with AD and mild cognitive impairment, and that combined exercise showed superior effects compared with single-type exercise, suggesting that the choice of intervention modality strongly influences sleep outcomes (25). Furthermore, a meta-analysis by Páez et al. confirmed that exercise has moderate-to-high-quality evidence supporting its benefits for both subjective sleep quality and objective sleep measures in patients with AD and Alzheimer’s disease-related dementias (ADRD), providing a more solid empirical basis for exercise intervention as a non-pharmacological adjunctive strategy (26).

Although several systematic reviews have investigated the effects of exercise on sleep quality among older adults or patients with mild cognitive impairment, significant methodological limitations still exist in the existing literature (27). On the one hand, the use of sleep assessment tools lacks consistency, with both subjective questionnaires and objective monitoring measures being applied, which results in poor comparability across different studies (28). On the other hand, exercise intervention protocols are not standardized in terms of type, intensity, and duration (29). In addition, the small sample sizes and high risk of bias in most studies make it difficult to draw consistent conclusions (30).

Current findings from primary studies are inconsistent, and detailed analyses of key variables such as exercise type, duration, and frequency are lacking. Therefore, building on previous research, the present study is the first to focus on this specific AD population. Using systematic review and meta-analysis, we comprehensively synthesize randomized controlled trials and further explore the moderating effects of exercise session duration, exercise type, and other variables on sleep outcomes. This study aims to provide evidence-based support for the clinical development of individualized exercise prescriptions.

## 2 Methods

This study adopted a research design of systematic review and meta-analysis, strictly adhering to the guidelines of the Preferred Reporting Items for Systematic Reviews and Meta-Analyses 2020 (55). The study protocol has been registered on the International Prospective Register of Systematic Reviews (PROSPERO: CRD420251270397) to ensure the transparency of the research process and the methodological rigor of the study.

### 2.1 Databases and search strategy

A systematic literature search was conducted for eligible studies published on or before December 31, 2025, across six databases, namely PubMed, the Cochrane Library, Embase, Web of Science, Scopus, and the China National Knowledge Infrastructure (CNKI). The search results from all databases underwent systematic screening, and the literature search was primarily performed using a strategy combining Medical Subject Headings (MeSH) with free-text terms. In addition, the reference lists of relevant articles were manually searched to supplement additional studies for this project. All analyses in this study were based on published literature, and thus ethical approval and informed consent from patients were not required. The search strategy is shown in Appendix 1.

### 2.2 Eligibility criteria for inclusion

1 Population: Participants were diagnosed with Alzheimer’s disease at baseline, were over 18 years of age, and there were no gender restrictions. The diagnosis of AD must be made according to well-established criteria, including the National Institute of Neurological and Communicative Disorders and Stroke–Alzheimer’s Disease and Related Disorders Association (NINCDS-ADRDA) criteria (31), and other formal medical diagnostic methods reported in the workgroups or papers of the National Institute on Aging–Alzheimer’s Association Guidelines for the Diagnosis of Alzheimer’s Disease (32).
2 Intervention: Exercise interventions of any type, frequency, and with a duration of no less than 4 weeks were included.
3 Control group: Studies with a control group receiving alternative interventions, such as routine care and stretching exercises.
4 Outcomes: The primary outcome measures were sleep-related indicators in AD patients, including sleep quality, sleep efficiency, and total sleep duration, among others. All indicators were continuous data obtained via objective measurements (e.g., actigraphy) or self-reported scales (e.g., the Pittsburgh Sleep Quality Index [PSQI]).
5 Study design: Only randomized controlled trials (RCTs) were included.

### 2.3 Exclusion criteria

1 Articles including comments, letters, case reports, conference abstracts, review articles, dissertations and unpublished manuscripts were excluded.
2 Studies from which valid outcome data could not be extracted from the literature, and no relevant data were obtained despite contacting the authors, were excluded.
3 Articles that were inaccessible and for which the full text could not be obtained through various channels were excluded.
4 Duplicate published articles were excluded.
5 Studies combining exercise interventions with dietary control or other lifestyle interventions were excluded.

### 2.4 Literature screening and data extraction

Literature screening and data extraction were independently conducted by two researchers. In case of discrepancies during the screening process, the researchers first attempted to reach a consensus through discussion, if a unanimous agreement could not be reached after discussion, the opinion of the third author was consulted to ensure the objectivity and impartiality of the evaluation process. Literature management and data extraction were performed using Endnote X9 and Excel software, respectively. For literature with missing data, the corresponding authors of the original articles were contacted to obtain complete information. The extracted literature information mainly included authors, year of publication, country/region, sample size, intervention type, intervention frequency, intervention duration, and outcome measures. Where no standard deviations (SDs) were available, they were calculated from standard errors (SEs), CIs, t or *p* values, or attempts were made to obtain the missing data from the authors by email. If the author did not report the data in the paper but provided a graph with the data, we used GetData Digitizer version 2.20 software to extract data we need from graphs.

### 2.5 Assessment of study quality

Two researchers independently assessed the risk of bias of the included studies in accordance with the Cochrane Handbook’s risk of bias assessment tool for RCTs, covering selection bias, performance bias, detection bias, attrition bias, reporting bias and other sources of bias. Quality evaluation was conducted using three grading levels: unclear, low risk and high risk, and the literature quality was categorized into high, moderate and low quality. In case of discrepancies, discussions were held or the opinion of a third researcher was sought.

### 2.6 Quality of evidence

This study assessed the strength of evidence for each outcome according to the Grading of Recommendations Assessment, Development, and Evaluation (GRADE) guidelines. Since all included studies were randomized controlled trials (RCTs), we systematically evaluated the quality based on the five downgrading criteria in the GRADE framework, including risk of bias, inconsistency, indirectness, imprecision, and publication bias, and determined whether to downgrade the level of evidence accordingly. Finally, the certainty of the evidence was categorized into four levels: high, moderate, low, and very low.

### 2.7 Data analysis

To minimize the impact of baseline differences on the results, this study pooled the effect sizes using the changes in post-intervention mean values and standard deviations. Data analysis was performed with Review Manager 5.4 software. The outcome measures in this study were continuous variables. Given the inconsistent measurement methods of outcome measures across the included studies, the standardized mean difference (SMD) was adopted for effect size pooling, with a *p*-value<0.05 considered statistically significant. Heterogeneity was comprehensively assessed using the homogeneity test (Q-test, significance level *α* = 0.10) and the I^2^ statistic. A negligible level of heterogeneity across studies was deemed to exist when *p* > 0.1 and I^2^ < 50%, in which case the fixed-effect model was used for pooling, significant heterogeneity was considered present when *p* < 0.10 and I^2^ > 50%, and the random-effect model was thus applied for pooling. For cases with significant heterogeneity, further sensitivity analysis, subgroup analysis, and univariate meta-regression were conducted to explore the potential sources of such heterogeneity. Funnel plots were constructed to assess publication bias for outcome measures with more than 10 included studies. The significance level was set at *α* = 0.05.

## 3 Results

**Figure 1:**
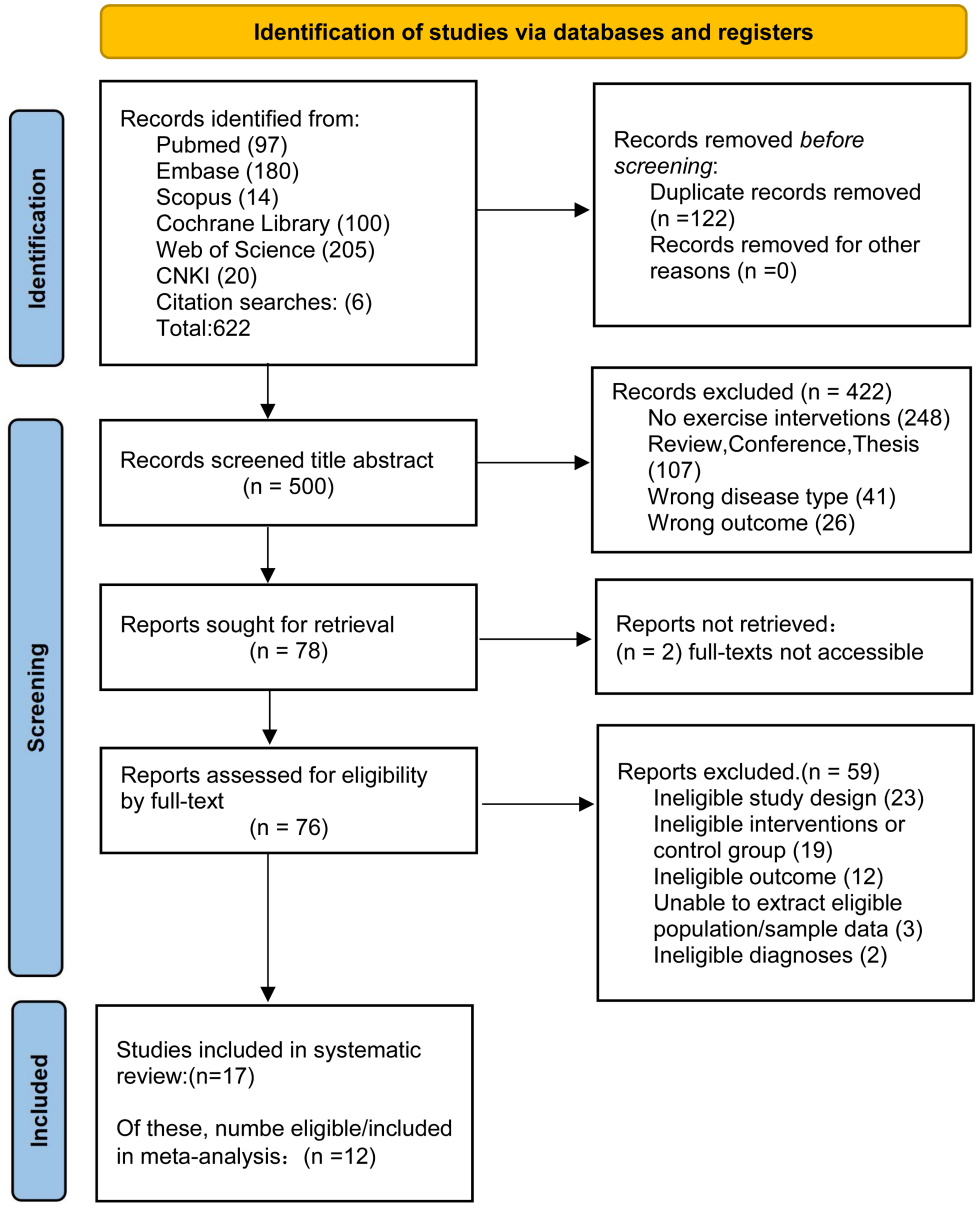
Study flow diagram.

A total of 616 records were retrieved from six databases in this study, with an additional 6 records supplemented from relevant journals and reference lists, amounting to 622 records in total. Duplicated literatures (*n* = 122) were identified and excluded using EndNote X9 software, leaving 500 records for the screening stage. Two researchers independently conducted a tiered screening based on titles, abstracts and full texts, resulting in the exclusion of 422 records overall. At the full-text screening stage, a total of 59 studies were excluded for failing to meet the inclusion criteria, with the specific reasons as follows: inconsistent study design (*n* = 23), inconsistent intervention measures (*n* = 19), inconsistent outcome measures (*n* = 12), inability to extract valid data (*n* = 3), and inconsistent diagnostic criteria (*n* = 2). A total of 17 randomized controlled trials (RCTs) were finally included, among which 12 met the criteria for effect size pooling in the meta-analysis. The entire literature screening process was conducted in accordance with the PRISMA guidelines, and the flow is presented in Figure 1.

### 3.1 Study characteristics

A total of 12 randomized controlled trials were included in this study, involving participants from 6 countries, among which the largest number of literatures were from China (*n* = 7). The total sample size of the studies was 893 participants, with the proportion of females exceeding 50% in most studies. The participants were mainly aged between 70 and 85 years, with a mean age of approximately 73–78 years. Diverse types of exercise interventions were adopted, including comprehensive training (*n* = 3), aerobic walking (*n* = 3), sport stacking (*n* = 2), resistance training (*n* = 1), yoga (*n* = 1), dance (*n* = 1), and Tai Chi Qigong (*n* = 1). The single session duration of interventions ranged from 20 to 90 min, the intervention periods varied from 6 weeks to 12 months, and the exercise frequency was 2 to 6 sessions per week. Sleep-related outcomes were primarily assessed using the Pittsburgh Sleep Quality Index (PSQI), actigraphy, and the Neuropsychiatric Inventory (NPI). The basic information of the included literatures is presented in Table 1.

**Table 1:** Basic information of the included literatures.

Study characteristics		Participant characteristics			Exercise characteristics				Outcome measurement
Author, year	Location	Sample size	Age Mean (SD)	% Female	Type of Exercise	Duration	Frequency	Length	Assessment type
Song et al. (37)	China	I: 45 C: 44	I:76.71 (5.96) C:75.20 (6.63)	I: 80C: 70	Aerobic Dancing	60 min	3 times/ week	16 weeks	PSQI
Jiang et al. (41)	China	I: 24C: 24	I:74.08 (6.12) C:72.67 (5.32)	I: 58.3C: 54.2	Cup stacking	At least 30 min	At least 5 days/week	6 months	PSQI
Yang et al. (40)	China	I1:9 C1:11 I2:9 C2: 10	I1:70.5 (5.4) C1:72.0 (8.1) I2:73.1 (5.2) C2:75.9 (8.0)	I1:63.6 C1:44.4 I2:80 C2:77.8	sport stacking	At least 30 min	5times/ weeks	12 weeks	PSQI
Li et al. (50)	China	I:22C:19	\	\	multi-component exercise	60 min	3times/ weeks	12 weeks	Actigraphy
Song and Yu (38)	China	I:60 C:60	I:76.22 (5.75) C:75.33 (6.78)	I: 80 C: 70	Aerobic Stepping	60 min	3times/ week	16 weeks	PSQI
Bademli et al. (36)	Turkey	I:30 C:30	I:72.24 (7.16) C:70.67 (8.34)	I:60C:56.7	multi-component exercise	80 min	3-4times/ weeks	20 weeks	PSQI
Mu et al. (35)	China	I:36C:36	I:73.8 (5.3) C:73.7 (5.1)	I:58.3C: 61.1	Brisk walking	20–30 min	4-6times/ weeks	12 weeks	PSQI
Öhman et al. (51)	Finland	I: 57 I: 63 C: 59	\	\	multi-component exercise	60 min	2 times/ week	12 months	NPI
Karydaki et al. (39)	Greece	I1: 15 C1: 9 I2: 16 C2: 9	\	\	Resistance Training Chair Yoga	45 min	2 times/ week	12 weeks	PSQI
Chan et al. (52)	China	I:25C:27	I:78.4 (7.1) C:82.2 (6.7)	I: 68 C: 100	Tai chi qigong	60 min	2times/ week	Short 2 months	PSQI
Eggermont et al. (53)	Netherlands	I: 41C: 38	\	\	Walking	30 min	5 times/ week	6 weeks	Actigraphy
McCurry et al. (54)	United States	I:32C: 33	I:82.2 (8.5) C:81.2 (8.0)	I: 53C: 51	Walking	At least 30 min	High daily	6 months	Actigraphy

RCT, Randomized controlled trial; AD, Alzheimer’s disease; I, intervention; C, control; PSQI, Pittsburgh sleep quality index.

### 3.2 Literature quality assessment

**Figure 2:**
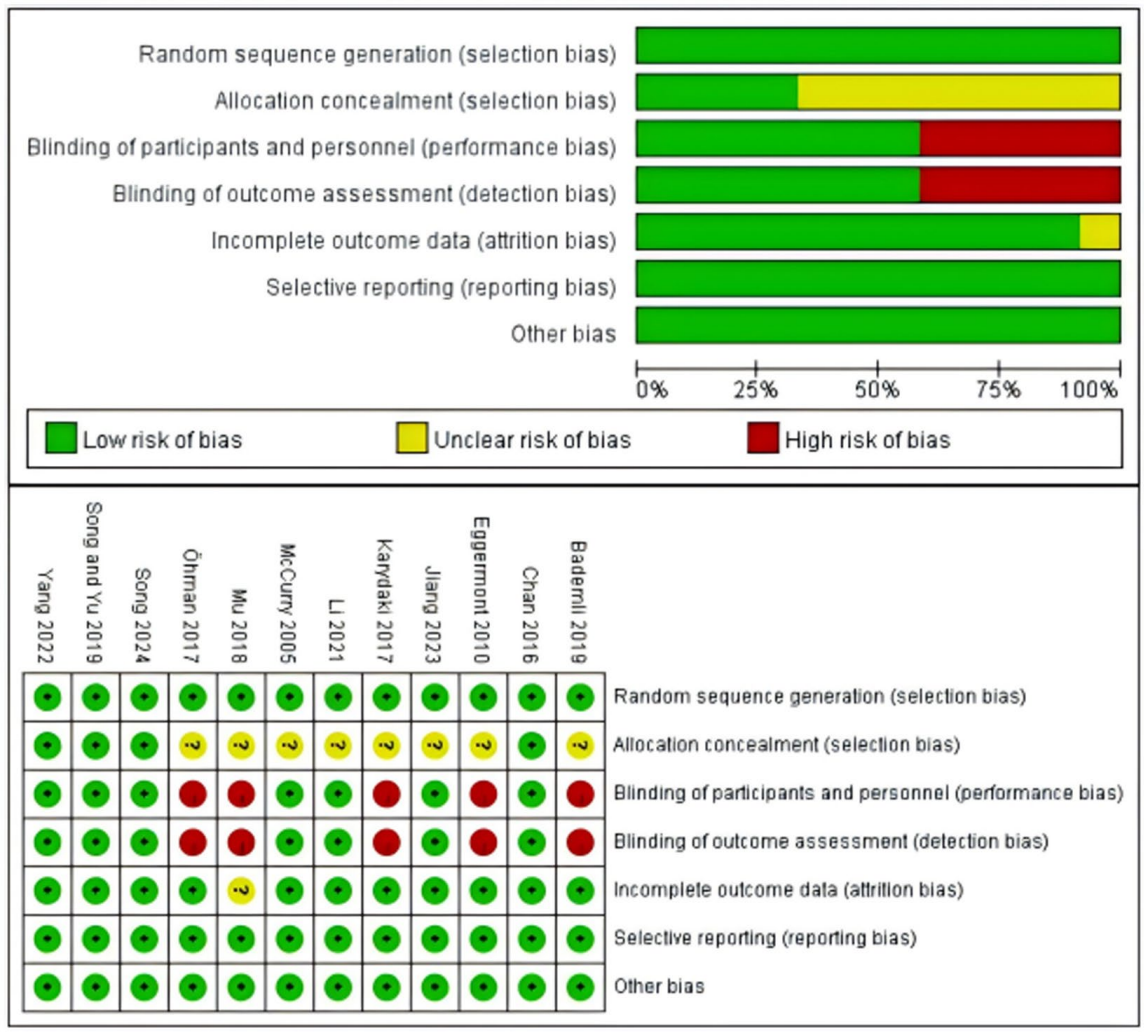
Risk of Bias Plot.

Twelve randomized controlled trials were included for the Cochrane Risk of Bias assessment. All the included studies provided a detailed report on the baseline characteristics of the participants, as well as specific descriptions of the intervention measures and evaluation indicators. Meanwhile, four studies elaborated on the specific grouping methods in detail. In addition, seven studies gave a comprehensive illustration of the application of blinding methods. The quality of the included studies is presented in Figure 2.

### 3.3 PSQI scores

**Figure 3:**
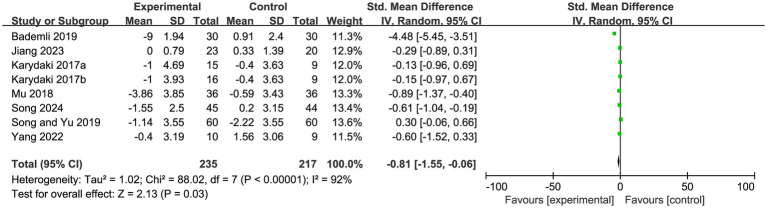
Meta-analysis of PSQI scores in patients with Alzheimer’s disease after exercise intervention.

A total of 8 articles focusing on the sleep quality of AD patients were included in the PSQI scale analysis. The analysis results showed that the combined effect of the experimental group on PSQI scores was statistically significant compared with the control group (SMD = −0.81, 95%CI: −1.55 to −0.06, Z = 2.13, *p* = 0.030). However, there was high heterogeneity among the included studies (I^2^ = 92, *p* < 0.001) (Figure 3). To address this issue, a sensitivity analysis was performed using Stata 15.0. After excluding the study by Bademil from the 8 studies on PSQI, the I^2^ value decreased by 23 to 69% (*p* = 0.004), while the overall effect test failed to reach statistical significance (Z = 1.680, *p* = 0.090). The quality of evidence for this outcome was rated as low.

According to the subgroup analyses, the effect of exercise interventions on PSQI scores in patients with AD varied to some extent across different stratified characteristics, but the overall direction of effect was consistent, suggesting that exercise interventions are generally beneficial for improving sleep quality.

**Figure 4:**
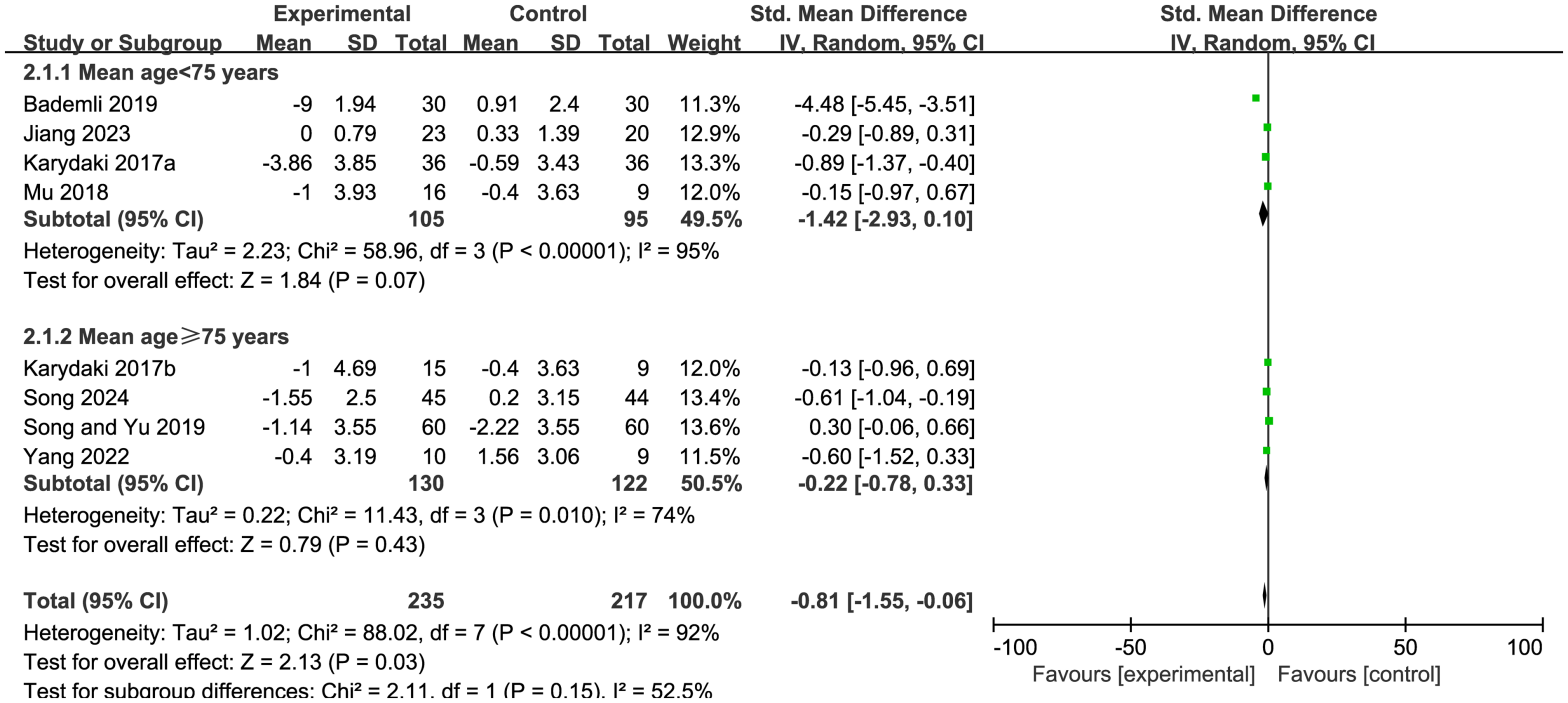
Subgroup analysis of PSQI scores by age in patients with Alzheimer’s disease.

When stratified by age, the subgroup with a mean age of <75 years showed an effect size of SMD = −1.42 (95% CI, −2.93 to 0.10, Z = 1.84, *p* = 0.07), indicating a relatively large trend toward improvement, although the difference did not reach statistical significance. In the subgroup with a mean age of ≥75 years, the effect size was SMD = −0.22 (95% CI, −0.78 to 0.33, Z = 0.79, *p* = 0.43), which was also not statistically significant. Although the direction of effect was consistent across the two age subgroups, some differences in effect magnitude were observed (Figure 4).

**Figure 5:**
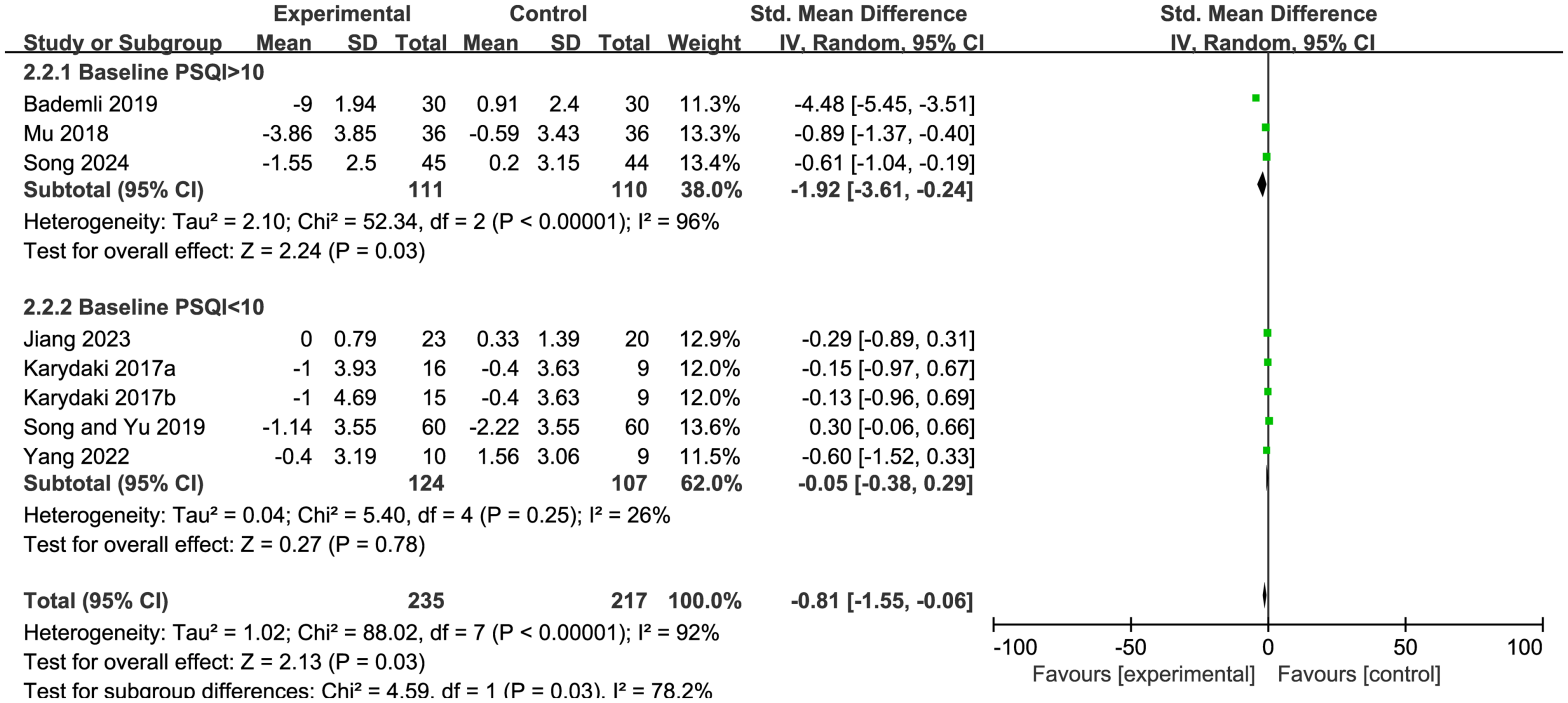
Subgroup analysis of PSQI scores by baseline PSQI in patients with Alzheimer’s disease.

When stratified by baseline sleep quality, the subgroup with a baseline PSQI score of >10 demonstrated a statistically significant improvement, with an effect size of SMD = −1.92 (95% CI, −3.61 to −0.24, Z = 2.24, *p* = 0.03). In contrast, the subgroup with a baseline PSQI score of <10 showed an effect size of SMD = −0.05 (95% CI, −0.38 to 0.29, Z = 0.27, *p* = 0.78), indicating no statistically significant difference. A marked difference in effect size was observed between the subgroups with different baseline sleep quality levels (Figure 5).

**Figure 6:**
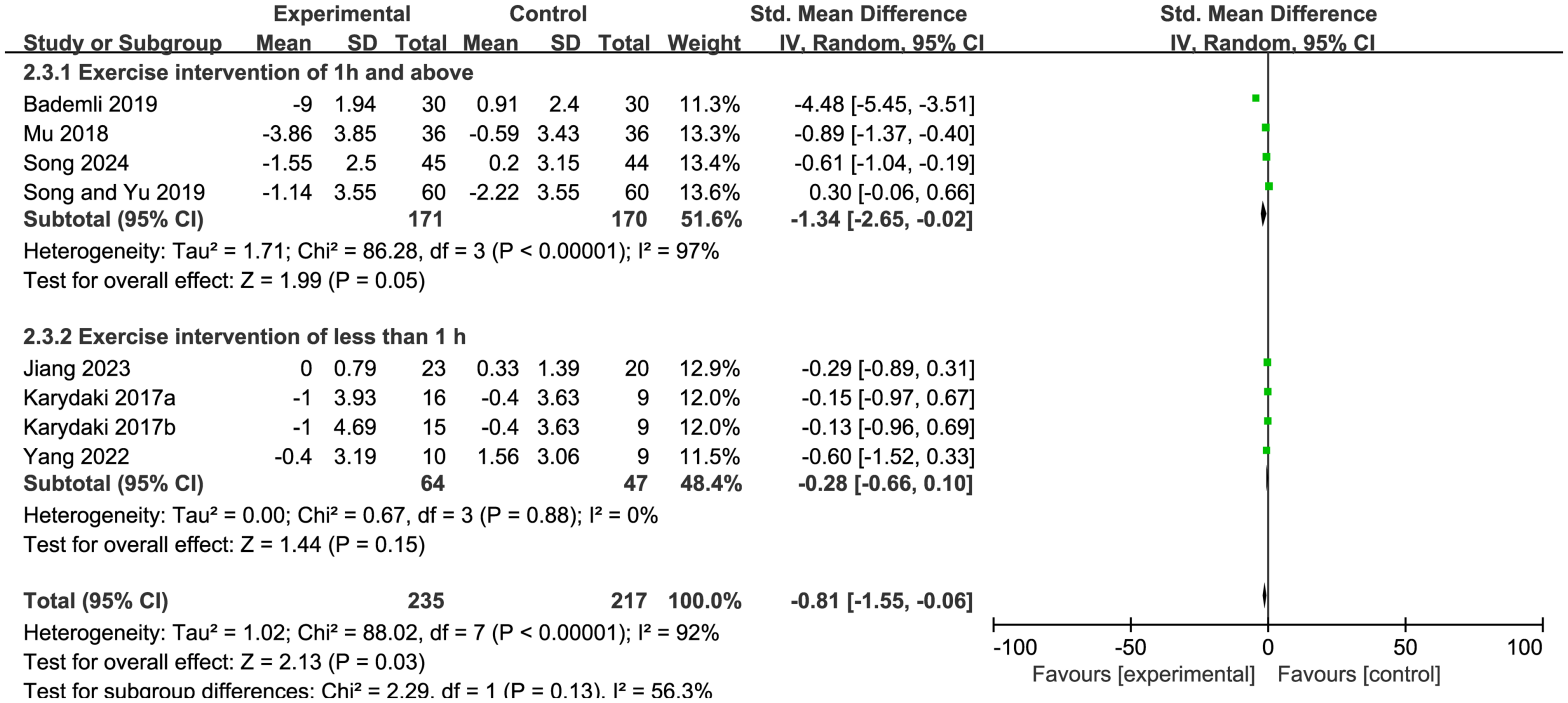
Subgroup analysis of PSQI scores by single exercise duration in patients with Alzheimer’s disease.

When stratified by the duration of each exercise session, studies with an intervention duration of ≥1 h per session showed a statistically significant improvement, with an effect size of SMD = −1.34 (95% CI: −2.65 to −0.02, Z = 1.99, *p* = 0.05). By contrast, studies with an intervention duration of <1 h per session yielded an effect size of SMD = −0.28 (95% CI: −0.66 to 0.10, Z = 1.44, *p* = 0.15), which did not reach statistical significance (Figure 6).

**Figure 7:**
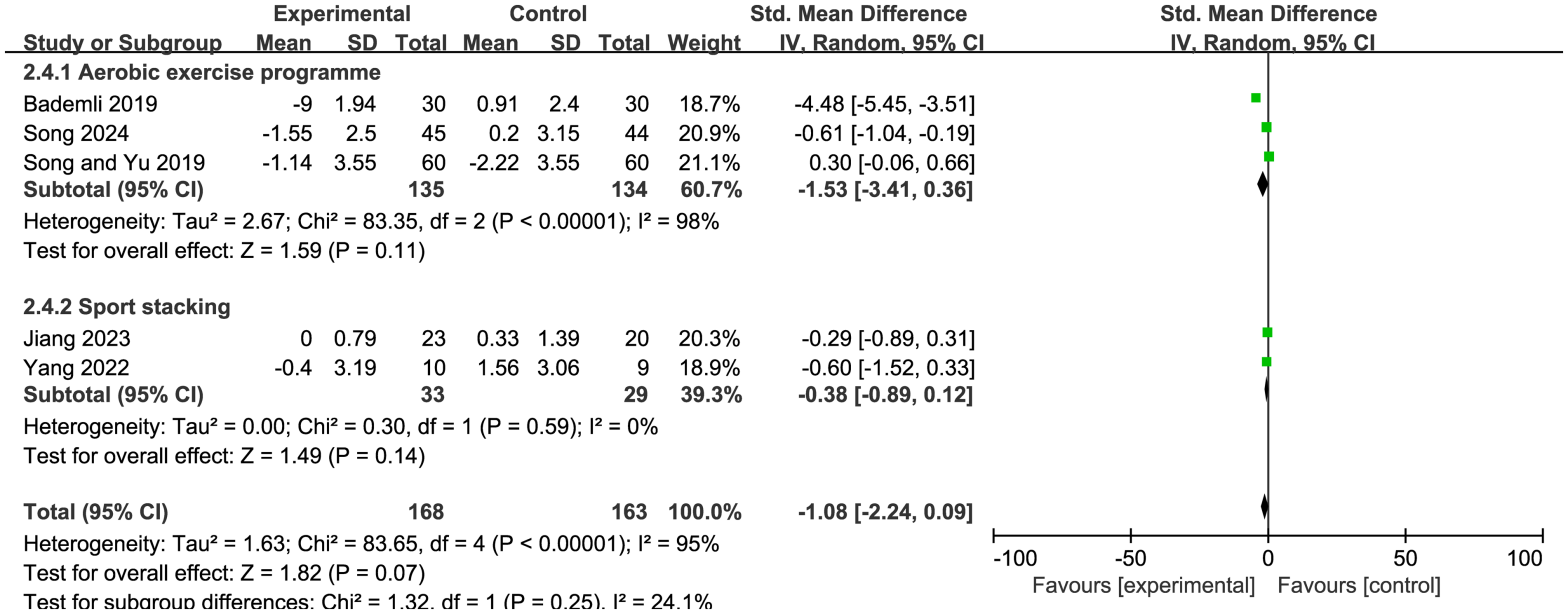
Subgroup analysis of PSQI scores by exercise type in patients with Alzheimer’s disease.

In the subgroup analysis by exercise type, the included exercise interventions were classified according to the predominant training characteristics of each program. Interventions primarily characterized by rhythmic large-muscle activity and cardiorespiratory endurance training were categorized as the aerobic exercise group (33), whereas interventions mainly characterized by hand-eye coordination, reaction ability, and fine motor practice were categorized as the sport stacking group (34). The results showed that the aerobic exercise group had an effect size of SMD = −1.34 (95% CI: −2.65 to −0.02, Z = 1.99, *p* = 0.05), reaching statistical significance. In contrast, the sport stacking group had an effect size of SMD = −0.38 (95% CI: −0.89 to 0.12, Z = 1.49, *p* = 0.14), which was not statistically significant (Figure 7).

### 3.4 Sleep efficiency

**Figure 8:**
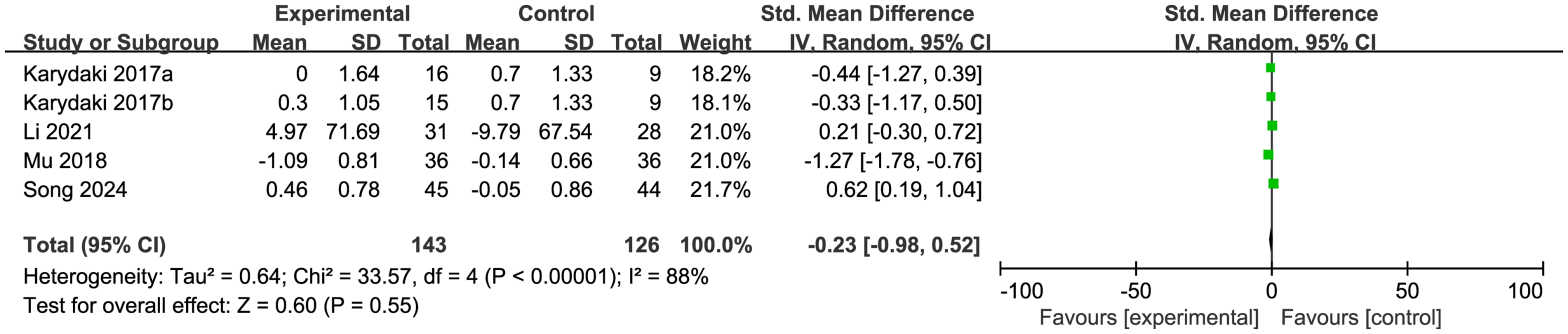
Meta-analysis of sleep efficiency in patients with Alzheimer’s disease after exercise intervention.

A total of 5 studies were included in this research to compare the difference in sleep duration between the experimental group and the control group, with a total sample size of 143 cases in the experimental group and 126 cases in the control group. The pooled effect between the intervention group and the control group was not statistically significant (SMD = −0.23, 95%CI: −0.98 to 0.52, Z = 0.60, *p* = 0.850), and there was high heterogeneity across the included studies (I^2^ = 88%, *p* < 0.001) (Figure 8). To address this issue, a sensitivity analysis was conducted using Stata 15.0. After excluding the study by Mu, the I^2^ value was 81% with a *p*-value of 0.01, indicating no obvious reduction in heterogeneity. This suggested that the results of this subgroup were greatly affected by other potential factors and lacked sufficient stability. The quality of evidence for this outcome was rated as very low.

### 3.5 Sleep duration

**Figure 9:**
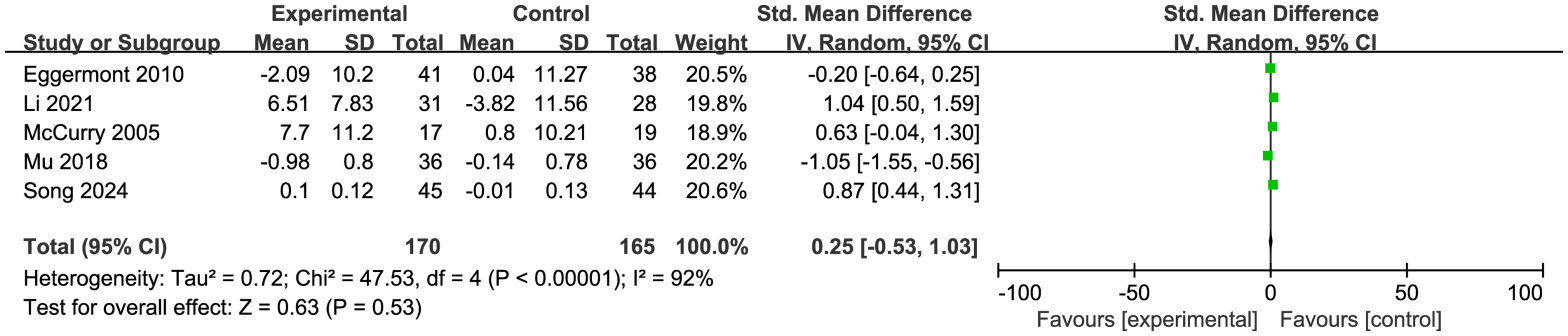
Meta-analysis of sleep duration in patients with Alzheimer’s disease after exercise intervention.

A total of 5 studies were included in this research to compare the difference in sleep efficiency between the experimental group and the control group, with a total sample size of 170 cases in the experimental group and 165 cases in the control group. The pooled effect between the intervention group and the control group was not statistically significant (SMD = 0.25, 95%CI: −0.53 to 1.03, Z = 0.63, *p* = 0.530), and there was high heterogeneity across the included studies (I^2^ = 92%, *p* < 0.001) (Figure 9). To address this issue, a sensitivity analysis was performed using Stata 15.0. After excluding the study by Mu, the I^2^ value dropped to 60% with a *p*-value of 0.06. Although the heterogeneity was close to the critical value, it remained at a moderate level, indicating that the consistency of the results was limited. The quality of evidence for this outcome was rated as very low.

### 3.6 Results of publication bias

**Figure 10:**
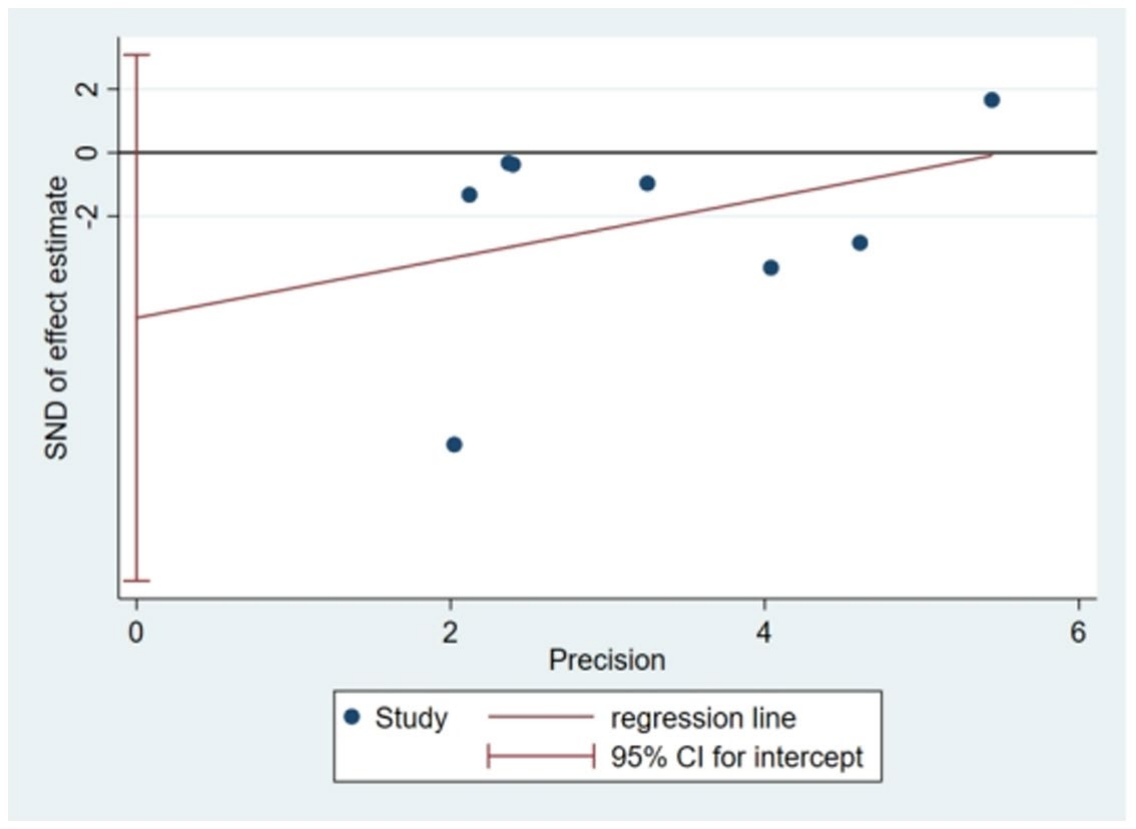
Publication Bias Plot.

Egger’s bias test was performed using Stata 15.0 in this study, yielding a *p*-value of 0.175 (*p* > 0.05). This result indicated that there was no publication bias among the selected literatures (Figure 10).

## 4 Discussion

The results of the meta-analysis indicated that exercise intervention could significantly improve the subjective sleep quality of patients with Alzheimer’s disease, with a pooled effect size of standardized mean difference (SMD) at −0.81 (95%CI: −1.55 to −0.06), which was statistically significant. Although the effect size was moderate, its clinical significance should not be overlooked, suggesting that regular exercise may serve as an effective non-pharmacological strategy to improve sleep perception and experience in AD patients. However, in terms of objective sleep indicators, this study found no significant improvement in sleep efficiency and total sleep duration brought by exercise, and a high level of heterogeneity was observed across all analyses. Sensitivity analysis revealed that individual studies had a substantial impact on the overall results, after their exclusion, the statistical significance of some results disappeared or heterogeneity only decreased moderately, indicating that the stability of the existing evidence is not yet sufficient.

Subgroup analyses in this study provided exploratory insights into potential factors that may influence the effects of exercise interventions on sleep outcomes in patients with AD. Patients with higher baseline PSQI scores appeared to derive greater benefit, suggesting that those with more severe baseline sleep disturbances may have greater room for improvement (35–37). Studies with a single-session intervention duration of at least 1 h also showed relatively greater improvements, indicating that a sufficient exercise dose per session may be important for sleep benefits (35–38). In contrast, although age-based and exercise-type subgroup analyses showed some differences in effect sizes, the between-subgroup differences were not statistically significant, suggesting that the current evidence is insufficient to support age or specific exercise modality as definitive effect modifiers (39–41). Overall, these subgroup findings suggest that the effects of exercise interventions may be jointly influenced by multiple factors. In addition to the stratification variables examined here, other factors, such as the stage of AD, the intervention setting, and differences in control conditions, may also contribute to the observed variability (42, 43).

Compared with previous systematic reviews on the elderly or individuals with mild cognitive impairment (MCI), this study strictly restricted the included participants to patients with a definitive diagnosis of AD, thereby avoiding bias caused by population heterogeneity (26, 44). Second, this study is the first to conduct a subgroup analysis on single-session exercise duration and found that interventions lasting ≥1 h yielded more significant effects, providing more actionable dosage recommendations for prescribing exercise in patients with AD. Compared with earlier studies targeting populations with mild cognitive impairment (MCI) and Alzheimer’s disease-related dementias (ADRD), this study further clarifies the population specificity and dose-dependency of exercise effects, providing a basis for the design of precise interventions in the future (25, 27).

Numerous studies have confirmed that exercise can effectively improve sleep quality in patients with AD and related cognitive disorders. As a safe non-pharmacological intervention, exercise can simultaneously improve sleep, cognition, and physical function in these patients (25, 26). Results from randomized controlled trials have demonstrated that exercise intervention significantly reduces the Pittsburgh Sleep Quality Index (PSQI) score in individuals with cognitive impairment, as reflected by shortened sleep latency, fewer nocturnal awakenings, prolonged total sleep time, and enhanced sleep efficiency (45). Improvements in sleep efficiency are directly associated with enhanced daytime function and quality of life (46). Although prior studies have suggested that baseline sleep quality may moderate the cognitive or sleep-related response to exercise, with greater improvements potentially observed in individuals with poorer initial sleep status, these findings remain inconclusive (47). Likewise, the potential superiority of multicomponent exercise over single-modality interventions has been reported in some studies (48). However, such evidence has largely been derived from individual studies, specific samples, or reviews including broader populations with cognitive impairment, such as MCI and ADRD, rather than patients with Alzheimer’s disease alone (48, 49). Given the heterogeneity in study populations, intervention characteristics, and outcome assessments, these findings cannot be directly extrapolated to the present meta-analysis. Accordingly, our results do not support a direct inference that poorer baseline sleep quality predicts greater benefit from exercise, nor do they establish the superiority of multicomponent exercise over single-modality exercise in AD patients.

The significance of this study lies in providing more disease-specific evidence for the role of exercise in improving sleep in patients with Alzheimer’s disease. Unlike previous studies that included individuals with MCI or other cognitive disorders, this study focused exclusively on patients with a confirmed diagnosis of AD, thereby enhancing the specificity of the findings. The results suggest that exercise may improve subjective sleep quality in AD patients, and that baseline sleep disturbance severity and exercise session duration may influence the intervention effect, providing preliminary support for individualized exercise prescription. Although evidence for objective sleep outcomes remains limited, this study still supports exercise as a non-pharmacological strategy for sleep management in AD and helps guide future high-quality research.

In addition, this study has certain limitations. Although the number and quality of included randomized controlled trials are within an acceptable range, the total sample size remains relatively limited. Moreover, there are substantial variations among the included studies in terms of exercise protocols, control group settings, outcome measurement tools and diagnostic criteria for AD, which have led to significant heterogeneity and limited the interpretability of the pooled results. Secondly, most studies failed to implement adequate blinding of assessors or participants, which may have introduced performance bias and detection bias. Furthermore, the insufficient number of studies investigating objective sleep indicators via actigraphy may have compromised the ability to capture the objective changes in sleep architecture.

Based on existing evidence, future studies should conduct large-sample, methodologically rigorous randomized controlled trials. Such trials should adopt standardized reporting guidelines for exercise interventions, and combine objective monitoring tools such as polysomnography with subjective rating scales to comprehensively assess sleep quality. In addition, subsequent research is needed to systematically compare the differential effects of various exercise types on different sleep parameters, and include long-term follow-up to clarify the sustained long-term effects of exercise on sleep and cognitive function in patients with AD, so as to provide high-level evidence-based support for the development and optimization of clinical exercise programs.

## 5 Conclusion

Exercise interventions can significantly improve subjective sleep quality in patients with Alzheimer’s disease, but no significant effects have been observed on objective sleep outcomes, such as sleep efficiency and total sleep duration. Patients with more severe baseline sleep disturbances and those receiving exercise sessions lasting at least 1 h may derive greater benefits from the intervention. Overall, exercise may serve as an effective adjunct or alternative to pharmacological treatment and can be incorporated into comprehensive care plans for patients with AD, thereby helping to alleviate sleep disturbances and reduce medication-related risks.

## Data Availability

The datasets presented in this study can be found in online repositories. The names of the repository/repositories and accession number(s) can be found in the article/Supplementary material.
