# Supplementary material for: Exercise intervention on sleep quality in Alzheimer’s disease: a systematic review and meta-analysis
Source: Front Med (Lausanne). 2026 May 8;13:1796892. doi: 10.3389/fmed.2026.1796892 (PMC13193829; doi:10.3389/fmed.2026.1796892)
Supplement: Supplementary file 1 [file Table_1.docx]

Appendix 1 Search strategy in PubMed

| Step | Search Strategy |
| --- | --- |
| #1 | "alzheimer disease"[Title/Abstract] OR ("alzheimer syndrome"[Title/Abstract] OR (("alzheimer disease"[MeSH Terms] OR ("Alzheimer"[All Fields] AND "Disease"[All Fields]) OR "alzheimer disease"[All Fields] OR ("Alzheimer"[All Fields] AND "Type"[All Fields] AND "Dementia"[All Fields]) OR "alzheimer type dementia"[All Fields]) AND "ATD"[Title/Abstract]) OR (("alzheimer disease"[MeSH Terms] OR ("Alzheimer"[All Fields] AND "Disease"[All Fields]) OR "alzheimer disease"[All Fields] OR ("Alzheimer"[All Fields] AND "Type"[All Fields] AND "Dementia"[All Fields]) OR "alzheimer type dementia"[All Fields]) AND "ATD"[Title/Abstract]) OR (("alzheimer disease"[MeSH Terms] OR ("Alzheimer"[All Fields] AND "Disease"[All Fields]) OR "alzheimer disease"[All Fields] OR ("Dementia"[All Fields] AND "Alzheimer"[All Fields] AND "Type"[All Fields]) OR "dementia alzheimer type"[All Fields]) AND "ATD"[Title/Abstract]) OR "alzheimer s diseases"[Title/Abstract] OR "alzheimer diseases"[Title/Abstract] OR "alzheimers diseases"[Title/Abstract] OR "alzheimer dementia"[Title/Abstract] OR "alzheimer dementias"[Title/Abstract] OR "dementia alzheimer"[Title/Abstract] OR "alzheimer s disease"[Title/Abstract] OR "dementia senile"[Title/Abstract] OR "senile dementia"[Title/Abstract] OR "dementia alzheimer type"[Title/Abstract] OR "alzheimer type dementia"[Title/Abstract] OR "senile dementia alzheimer type"[Title/Abstract] OR "alzheimer type senile dementia"[Title/Abstract] OR "primary senile degenerative dementia"[Title/Abstract] OR "alzheimer sclerosis"[Title/Abstract] OR "sclerosis alzheimer"[Title/Abstract] OR "dementia primary senile degenerative"[Title/Abstract] OR "dementia presenile"[Title/Abstract] OR "presenile dementia"[Title/Abstract] OR "acute confusional senile dementia"[Title/Abstract] OR (("alzheimer disease"[MeSH Terms] OR ("Alzheimer"[All Fields] AND "Disease"[All Fields]) OR "alzheimer disease"[All Fields] OR ("Senile"[All Fields] AND "Dementia"[All Fields]) OR "senile dementia"[All Fields]) AND "acute confusional"[Title/Abstract]) OR "alzheimer disease early onset"[Title/Abstract] OR "early onset alzheimer disease"[Title/Abstract] OR "presenile alzheimer dementia"[Title/Abstract] OR "alzheimer disease late onset"[Title/Abstract] OR "late onset alzheimer disease"[Title/Abstract] OR (("alzheimer disease"[MeSH Terms] OR ("Alzheimer"[All Fields] AND "Disease"[All Fields]) OR "alzheimer disease"[All Fields] OR ("Alzheimer's"[All Fields] AND "Disease"[All Fields]) OR "alzheimer s disease"[All Fields]) AND "focal onset"[Title/Abstract]) OR (("Focal"[All Fields] OR "focalities"[All Fields] OR "focality"[All Fields] OR "focalization"[All Fields] OR "focalized"[All Fields] OR "focally"[All Fields] OR "focals"[All Fields] OR "local"[All Fields] OR "localisation"[All Fields] OR "localisations"[All Fields] OR "localise"[All Fields] OR "localised"[All Fields] OR "localises"[All Fields] OR "localising"[All Fields] OR "localization"[All Fields] OR "localizations"[All Fields] OR "localize"[All Fields] OR "localized"[All Fields] OR "localizer"[All Fields] OR "localizers"[All Fields] OR "localizes"[All Fields] OR "localizing"[All Fields] OR "locally"[All Fields] OR "locals"[All Fields]) AND "onset alzheimer s disease"[Title/Abstract]) OR (("alzheimer disease"[MeSH Terms] OR ("Alzheimer"[All Fields] AND "Disease"[All Fields]) OR "alzheimer disease"[All Fields] OR ("familial"[All Fields] AND "Alzheimer"[All Fields] AND "Disease"[All Fields]) OR "familial alzheimer disease"[All Fields]) AND "FAD"[Title/Abstract]) OR ((("alzheimer disease"[MeSH Terms] OR ("Alzheimer"[All Fields] AND "Disease"[All Fields]) OR "alzheimer disease"[All Fields]) AND ("familialities"[All Fields] OR "familiality"[All Fields] OR "familially"[All Fields] OR "familials"[All Fields] OR "familie"[All Fields] OR "family"[MeSH Terms] OR "family"[All Fields] OR "familial"[All Fields] OR "families"[All Fields] OR "family s"[All Fields] OR "familys"[All Fields])) AND "FAD"[Title/Abstract]) OR ((("familialities"[All Fields] OR "familiality"[All Fields] OR "familially"[All Fields] OR "familials"[All Fields] OR "familie"[All Fields] OR "family"[MeSH Terms] OR "family"[All Fields] OR "familial"[All Fields] OR "families"[All Fields] OR "family s"[All Fields] OR "familys"[All Fields]) AND ("alzheimer disease"[MeSH Terms] OR ("Alzheimer"[All Fields] AND "Disease"[All Fields]) OR "alzheimer disease"[All Fields] OR ("Alzheimer"[All Fields] AND "Diseases"[All Fields]) OR "alzheimer diseases"[All Fields])) AND "FAD"[Title/Abstract])) |
| #2 | "exercise"[MeSH Terms] OR "exercise"[All Fields] OR "exercises"[All Fields] OR "exercise therapy"[MeSH Terms] OR ("exercise"[All Fields] AND "therapy"[All Fields]) OR "exercise therapy"[All Fields] OR "exercising"[All Fields] OR "exercise s"[All Fields] OR "exercised"[All Fields] OR "exerciser"[All Fields] OR "exercisers"[All Fields] OR ("exercise"[MeSH Terms] OR "exercise"[All Fields] OR "exercises"[All Fields] OR "exercise therapy"[MeSH Terms] OR ("exercise"[All Fields] AND "therapy"[All Fields]) OR "exercise therapy"[All Fields] OR "exercising"[All Fields] OR "exercise s"[All Fields] OR "exercised"[All Fields] OR "exerciser"[All Fields] OR "exercisers"[All Fields] OR ("exercise"[MeSH Terms] OR "exercise"[All Fields] OR ("exercise"[All Fields] AND "physical"[All Fields]) OR "exercise physical"[All Fields]) OR ("exercise"[MeSH Terms] OR "exercise"[All Fields] OR ("exercises"[All Fields] AND "physical"[All Fields]) OR "exercises physical"[All Fields]) OR ("exercise"[MeSH Terms] OR "exercise"[All Fields] OR ("physical"[All Fields] AND "exercise"[All Fields]) OR "physical exercise"[All Fields]) OR ("exercise"[MeSH Terms] OR "exercise"[All Fields] OR ("physical"[All Fields] AND "exercises"[All Fields]) OR "physical exercises"[All Fields]) OR ("exercise"[MeSH Terms] OR "exercise"[All Fields] OR ("exercise"[All Fields] AND "aerobic"[All Fields]) OR "exercise aerobic"[All Fields]) OR ("exercise"[MeSH Terms] OR "exercise"[All Fields] OR ("aerobic"[All Fields] AND "exercise"[All Fields]) OR "aerobic exercise"[All Fields]) OR ("exercise"[MeSH Terms] OR "exercise"[All Fields] OR ("aerobic"[All Fields] AND "exercises"[All Fields]) OR "aerobic exercises"[All Fields]) OR ("exercise"[MeSH Terms] OR "exercise"[All Fields] OR ("exercises"[All Fields] AND "aerobic"[All Fields]) OR "exercises aerobic"[All Fields]) OR ("exercise"[MeSH Terms] OR "exercise"[All Fields] OR ("exercise"[All Fields] AND "isometric"[All Fields]) OR "exercise isometric"[All Fields]) OR ("exercise"[MeSH Terms] OR "exercise"[All Fields] OR ("exercises"[All Fields] AND "isometric"[All Fields]) OR "exercises isometric"[All Fields]) OR ("exercise"[MeSH Terms] OR "exercise"[All Fields] OR ("isometric"[All Fields] AND "exercises"[All Fields]) OR "isometric exercises"[All Fields]) OR ("exercise"[MeSH Terms] OR "exercise"[All Fields] OR ("isometric"[All Fields] AND "exercise"[All Fields]) OR "isometric exercise"[All Fields]) OR ("exercise"[MeSH Terms] OR "exercise"[All Fields] OR ("acute"[All Fields] AND "exercise"[All Fields]) OR "acute exercise"[All Fields]) OR ("exercise"[MeSH Terms] OR "exercise"[All Fields] OR ("acute"[All Fields] AND "exercises"[All Fields]) OR "acute exercises"[All Fields]) OR ("exercise"[MeSH Terms] OR "exercise"[All Fields] OR ("exercise"[All Fields] AND "acute"[All Fields]) OR "exercise acute"[All Fields]) OR ("exercise"[MeSH Terms] OR "exercise"[All Fields] OR ("exercises"[All Fields] AND "acute"[All Fields]) OR "exercises acute"[All Fields]) OR ("exercise"[MeSH Terms] OR "exercise"[All Fields] OR ("exercise"[All Fields] AND "training"[All Fields]) OR "exercise training"[All Fields]) OR ("exercise"[MeSH Terms] OR "exercise"[All Fields] OR ("exercise"[All Fields] AND "trainings"[All Fields]) OR "exercise trainings"[All Fields]) OR ("exercise"[MeSH Terms] OR "exercise"[All Fields] OR ("training"[All Fields] AND "exercise"[All Fields]) OR "training exercise"[All Fields]) OR ("exercise"[MeSH Terms] OR "exercise"[All Fields] OR ("trainings"[All Fields] AND "exercise"[All Fields])) OR ("exercise"[MeSH Terms] OR "exercise"[All Fields] OR ("physical"[All Fields] AND "activity"[All Fields]) OR "physical activity"[All Fields]) OR ("exercise"[MeSH Terms] OR "exercise"[All Fields] OR ("activities"[All Fields] AND "physical"[All Fields]) OR "activities physical"[All Fields]) OR ("exercise"[MeSH Terms] OR "exercise"[All Fields] OR ("activity"[All Fields] AND "physical"[All Fields]) OR "activity physical"[All Fields]) OR ("exercise"[MeSH Terms] OR "exercise"[All Fields] OR ("physical"[All Fields] AND "activities"[All Fields]) OR "physical activities"[All Fields])) |
| #3 | "sleep quality"[MeSH Terms] OR ("sleep"[All Fields] AND "quality"[All Fields]) OR "sleep quality"[All Fields] OR ("sleep quality"[MeSH Terms] OR ("sleep"[All Fields] AND "quality"[All Fields]) OR "sleep quality"[All Fields] OR ("qualities"[All Fields] AND "sleep"[All Fields]) OR "qualities sleep"[All Fields]) OR ("sleep quality"[MeSH Terms] OR ("sleep"[All Fields] AND "quality"[All Fields]) OR "sleep quality"[All Fields] OR ("quality"[All Fields] AND "sleep"[All Fields]) OR "quality sleep"[All Fields]) OR ("sleep quality"[MeSH Terms] OR ("sleep"[All Fields] AND "quality"[All Fields]) OR "sleep quality"[All Fields] OR ("sleep"[All Fields] AND "qualities"[All Fields]) OR "sleep qualities"[All Fields]) |
| #4 | ("exercise"[MeSH Terms] OR "exercise"[All Fields] OR "exercises"[All Fields] OR "exercise therapy"[MeSH Terms] OR ("exercise"[All Fields] AND "therapy"[All Fields]) OR "exercise therapy"[All Fields] OR "exercising"[All Fields] OR "exercise s"[All Fields] OR "exercised"[All Fields] OR "exerciser"[All Fields] OR "exercisers"[All Fields] OR ("exercise"[MeSH Terms] OR "exercise"[All Fields] OR "exercises"[All Fields] OR "exercise therapy"[MeSH Terms] OR ("exercise"[All Fields] AND "therapy"[All Fields]) OR "exercise therapy"[All Fields] OR "exercising"[All Fields] OR "exercise s"[All Fields] OR "exercised"[All Fields] OR "exerciser"[All Fields] OR "exercisers"[All Fields] OR ("exercise"[MeSH Terms] OR "exercise"[All Fields] OR ("exercise"[All Fields] AND "physical"[All Fields]) OR "exercise physical"[All Fields]) OR ("exercise"[MeSH Terms] OR "exercise"[All Fields] OR ("exercises"[All Fields] AND "physical"[All Fields]) OR "exercises physical"[All Fields]) OR ("exercise"[MeSH Terms] OR "exercise"[All Fields] OR ("physical"[All Fields] AND "exercise"[All Fields]) OR "physical exercise"[All Fields]) OR ("exercise"[MeSH Terms] OR "exercise"[All Fields] OR ("physical"[All Fields] AND "exercises"[All Fields]) OR "physical exercises"[All Fields]) OR ("exercise"[MeSH Terms] OR "exercise"[All Fields] OR ("exercise"[All Fields] AND "aerobic"[All Fields]) OR "exercise aerobic"[All Fields]) OR ("exercise"[MeSH Terms] OR "exercise"[All Fields] OR ("aerobic"[All Fields] AND "exercise"[All Fields]) OR "aerobic exercise"[All Fields]) OR ("exercise"[MeSH Terms] OR "exercise"[All Fields] OR ("aerobic"[All Fields] AND "exercises"[All Fields]) OR "aerobic exercises"[All Fields]) OR ("exercise"[MeSH Terms] OR "exercise"[All Fields] OR ("exercises"[All Fields] AND "aerobic"[All Fields]) OR "exercises aerobic"[All Fields]) OR ("exercise"[MeSH Terms] OR "exercise"[All Fields] OR ("exercise"[All Fields] AND "isometric"[All Fields]) OR "exercise isometric"[All Fields]) OR ("exercise"[MeSH Terms] OR "exercise"[All Fields] OR ("exercises"[All Fields] AND "isometric"[All Fields]) OR "exercises isometric"[All Fields]) OR ("exercise"[MeSH Terms] OR "exercise"[All Fields] OR ("isometric"[All Fields] AND "exercises"[All Fields]) OR "isometric exercises"[All Fields]) OR ("exercise"[MeSH Terms] OR "exercise"[All Fields] OR ("isometric"[All Fields] AND "exercise"[All Fields]) OR "isometric exercise"[All Fields]) OR ("exercise"[MeSH Terms] OR "exercise"[All Fields] OR ("Acute"[All Fields] AND "exercise"[All Fields]) OR "acute exercise"[All Fields]) OR ("exercise"[MeSH Terms] OR "exercise"[All Fields] OR ("Acute"[All Fields] AND "exercises"[All Fields]) OR "acute exercises"[All Fields]) OR ("exercise"[MeSH Terms] OR "exercise"[All Fields] OR ("exercise"[All Fields] AND "Acute"[All Fields]) OR "exercise acute"[All Fields]) OR ("exercise"[MeSH Terms] OR "exercise"[All Fields] OR ("exercises"[All Fields] AND "Acute"[All Fields]) OR "exercises acute"[All Fields]) OR ("exercise"[MeSH Terms] OR "exercise"[All Fields] OR ("exercise"[All Fields] AND "training"[All Fields]) OR "exercise training"[All Fields]) OR ("exercise"[MeSH Terms] OR "exercise"[All Fields] OR ("exercise"[All Fields] AND "trainings"[All Fields]) OR "exercise trainings"[All Fields]) OR ("exercise"[MeSH Terms] OR "exercise"[All Fields] OR ("training"[All Fields] AND "exercise"[All Fields]) OR "training exercise"[All Fields]) OR ("exercise"[MeSH Terms] OR "exercise"[All Fields] OR ("trainings"[All Fields] AND "exercise"[All Fields])) OR ("exercise"[MeSH Terms] OR "exercise"[All Fields] OR ("physical"[All Fields] AND "activity"[All Fields]) OR "physical activity"[All Fields]) OR ("exercise"[MeSH Terms] OR "exercise"[All Fields] OR ("activities"[All Fields] AND "physical"[All Fields]) OR "activities physical"[All Fields]) OR ("exercise"[MeSH Terms] OR "exercise"[All Fields] OR ("activity"[All Fields] AND "physical"[All Fields]) OR "activity physical"[All Fields]) OR ("exercise"[MeSH Terms] OR "exercise"[All Fields] OR ("physical"[All Fields] AND "activities"[All Fields]) OR "physical activities"[All Fields]))) AND ("alzheimer disease"[Title/Abstract] OR ("alzheimer syndrome"[Title/Abstract] OR (("alzheimer disease"[MeSH Terms] OR ("Alzheimer"[All Fields] AND "Disease"[All Fields]) OR "alzheimer disease"[All Fields] OR ("Alzheimer"[All Fields] AND "Type"[All Fields] AND "Dementia"[All Fields]) OR "alzheimer type dementia"[All Fields]) AND "ATD"[Title/Abstract]) OR (("alzheimer disease"[MeSH Terms] OR ("Alzheimer"[All Fields] AND "Disease"[All Fields]) OR "alzheimer disease"[All Fields] OR ("Alzheimer"[All Fields] AND "Type"[All Fields] AND "Dementia"[All Fields]) OR "alzheimer type dementia"[All Fields]) AND "ATD"[Title/Abstract]) OR (("alzheimer disease"[MeSH Terms] OR ("Alzheimer"[All Fields] AND "Disease"[All Fields]) OR "alzheimer disease"[All Fields] OR ("Dementia"[All Fields] AND "Alzheimer"[All Fields] AND "Type"[All Fields]) OR "dementia alzheimer type"[All Fields]) AND "ATD"[Title/Abstract]) OR "alzheimer s diseases"[Title/Abstract] OR "alzheimer diseases"[Title/Abstract] OR "alzheimers diseases"[Title/Abstract] OR "alzheimer dementia"[Title/Abstract] OR "alzheimer dementias"[Title/Abstract] OR "dementia alzheimer"[Title/Abstract] OR "alzheimer s disease"[Title/Abstract] OR "dementia senile"[Title/Abstract] OR "senile dementia"[Title/Abstract] OR "dementia alzheimer type"[Title/Abstract] OR "alzheimer type dementia"[Title/Abstract] OR "senile dementia alzheimer type"[Title/Abstract] OR "alzheimer type senile dementia"[Title/Abstract] OR "primary senile degenerative dementia"[Title/Abstract] OR "alzheimer sclerosis"[Title/Abstract] OR "sclerosis alzheimer"[Title/Abstract] OR "dementia primary senile degenerative"[Title/Abstract] OR "dementia presenile"[Title/Abstract] OR "presenile dementia"[Title/Abstract] OR "acute confusional senile dementia"[Title/Abstract] OR (("alzheimer disease"[MeSH Terms] OR ("Alzheimer"[All Fields] AND "Disease"[All Fields]) OR "alzheimer disease"[All Fields] OR ("Senile"[All Fields] AND "Dementia"[All Fields]) OR "senile dementia"[All Fields]) AND "acute confusional"[Title/Abstract]) OR "alzheimer disease early onset"[Title/Abstract] OR "early onset alzheimer disease"[Title/Abstract] OR "presenile alzheimer dementia"[Title/Abstract] OR "alzheimer disease late onset"[Title/Abstract] OR "late onset alzheimer disease"[Title/Abstract] OR (("alzheimer disease"[MeSH Terms] OR ("Alzheimer"[All Fields] AND "Disease"[All Fields]) OR "alzheimer disease"[All Fields] OR ("Alzheimer's"[All Fields] AND "Disease"[All Fields]) OR "alzheimer s disease"[All Fields]) AND "focal onset"[Title/Abstract]) OR (("Focal"[All Fields] OR "focalities"[All Fields] OR "focality"[All Fields] OR "focalization"[All Fields] OR "focalized"[All Fields] OR "focally"[All Fields] OR "focals"[All Fields] OR "local"[All Fields] OR "localisation"[All Fields] OR "localisations"[All Fields] OR "localise"[All Fields] OR "localised"[All Fields] OR "localises"[All Fields] OR "localising"[All Fields] OR "localization"[All Fields] OR "localizations"[All Fields] OR "localize"[All Fields] OR "localized"[All Fields] OR "localizer"[All Fields] OR "localizers"[All Fields] OR "localizes"[All Fields] OR "localizing"[All Fields] OR "locally"[All Fields] OR "locals"[All Fields]) AND "onset alzheimer s disease"[Title/Abstract]) OR (("alzheimer disease"[MeSH Terms] OR ("Alzheimer"[All Fields] AND "Disease"[All Fields]) OR "alzheimer disease"[All Fields] OR ("familial"[All Fields] AND "Alzheimer"[All Fields] AND "Disease"[All Fields]) OR "familial alzheimer disease"[All Fields]) AND "FAD"[Title/Abstract]) OR ((("alzheimer disease"[MeSH Terms] OR ("Alzheimer"[All Fields] AND "Disease"[All Fields]) OR "alzheimer disease"[All Fields]) AND ("familialities"[All Fields] OR "familiality"[All Fields] OR "familially"[All Fields] OR "familials"[All Fields] OR "familie"[All Fields] OR "family"[MeSH Terms] OR "family"[All Fields] OR "familial"[All Fields] OR "families"[All Fields] OR "family s"[All Fields] OR "familys"[All Fields])) AND "FAD"[Title/Abstract]) OR ((("familialities"[All Fields] OR "familiality"[All Fields] OR "familially"[All Fields] OR "familials"[All Fields] OR "familie"[All Fields] OR "family"[MeSH Terms] OR "family"[All Fields] OR "familial"[All Fields] OR "families"[All Fields] OR "family s"[All Fields] OR "familys"[All Fields]) AND ("alzheimer disease"[MeSH Terms] OR ("Alzheimer"[All Fields] AND "Disease"[All Fields]) OR "alzheimer disease"[All Fields] OR ("Alzheimer"[All Fields] AND "Diseases"[All Fields]) OR "alzheimer diseases"[All Fields])) AND "FAD"[Title/Abstract]))) AND ("sleep quality"[MeSH Terms] OR ("sleep"[All Fields] AND "quality"[All Fields]) OR "sleep quality"[All Fields] OR ("sleep quality"[MeSH Terms] OR ("sleep"[All Fields] AND "quality"[All Fields]) OR "sleep quality"[All Fields] OR ("qualities"[All Fields] AND "sleep"[All Fields]) OR "qualities sleep"[All Fields]) OR ("sleep quality"[MeSH Terms] OR ("sleep"[All Fields] AND "quality"[All Fields]) OR "sleep quality"[All Fields] OR ("quality"[All Fields] AND "sleep"[All Fields]) OR "quality sleep"[All Fields]) OR ("sleep quality"[MeSH Terms] OR ("sleep"[All Fields] AND "quality"[All Fields]) OR "sleep quality"[All Fields] OR ("sleep"[All Fields] AND "qualities"[All Fields]) OR "sleep qualities"[All Fields])) |
